# Dexras1 links glucocorticoids to insulin-like growth factor-1 signaling in adipogenesis

**DOI:** 10.1038/srep28648

**Published:** 2016-06-27

**Authors:** Hyo Jung Kim, Jiyoung Y. Cha, Jo Woon Seok, Yoonjeong Choi, Bo Kyung Yoon, Hyeonjin Choi, Jung Hwan Yu, Su Jin Song, Ara Kim, Hyemin Lee, Daeun Kim, Ji Yoon Han, Jae-woo Kim

**Affiliations:** 1 Department of Biochemistry and Molecular Biology, Integrated Genomic Research Center for Metabolic Regulation, Institute of Genetic Science, Yonsei University College of Medicine, Seoul 120-752, Korea; 2 The Solomon H. Snyder Department of Neuroscience, Johns Hopkins University School of Medicine, Baltimore, MD 21205, USA; 3 Brain Korea 21 PLUS Project for Medical Science, Yonsei University, Seoul 120-752, Korea; 4 Department of Integrated OMICS for Biomedical Sciences, Graduate School, Yonsei University, Seoul 120-749, Korea

## Abstract

Glucocorticoids are associated with obesity, but the underlying mechanism by which they function remains poorly understood. Previously, we showed that small G protein Dexras1 is expressed by glucocorticoids and leads to adipocyte differentiation. In this study, we explored the mechanism by which Dexras1 mediates adipogenesis and show a link to the insulin-like growth factor-1 (IGF-1) signaling pathway. Without Dexras1, the activation of MAPK and subsequent phosphorylation of CCAAT/enhancer binding protein β (C/EBPβ) is abolished, thereby inhibiting mitotic clonal expansion and further adipocyte differentiation. Dexras1 translocates to the plasma membrane upon insulin or IGF-1 treatment, for which the unique C-terminal domain (amino acids 223–276) is essential. Dexras1-dependent MAPK activation is selectively involved in the IGF-1 signaling, because another Ras protein, H-ras localized to the plasma membrane independently of insulin treatment. Moreover, neither epidermal growth factor nor other cell types shows Dexras1-dependent MAPK activation, indicating the importance of Dexras1 in IGF-1 signaling in adipogenesis. Dexras1 interacts with Shc and Raf, indicating that Dexras1-induced activation of MAPK is largely dependent on the Shc-Grb2-Raf complex. These results suggest that Dexras1 is a critical mediator of the IGF-1 signal to activate MAPK, linking glucocorticoid signaling to IGF-1 signaling in adipogenesis.

Obesity is a major health concern with well-established links to diabetes, hypertension and cardiovascular disability^1^,^2^. Obesity is associated with increased adipocyte differentiation of precursor cells into mature adipocytes^3^,^4^,^5^. Cyclic AMP, insulin/insulin-like growth factor-1 (IGF-1), and glucocorticoid signaling pathway are all implicated in the adipocyte differentiation process^6^,^7^. To study adipocyte differentiation, 3T3-L1 preadipocytes are a commonly used cell line^8^. Upon hormonal stimulation with 1-methyl 3-isobutylxanthine (IBMX), dexamethasone, and insulin, growth-arrested 3T3-L1 preadipocytes re-enter the cell cycle synchronously. This process is known as a mitotic clonal expansion^6^. Once differentiated, these cells express adipocyte-specific genes for terminal differentiation^7^,^9^.

The signaling pathways for IBMX or insulin in adipogenesis have been extensively investigated over the past 40 years. Increased level of cAMP, for example, induces protein kinase A-mediated phosphorylation of cAMP response element-binding protein (CREB), which in turn promotes the expression of CCAAT/enhancer binding protein β (C/EBPβ)^10^. Insulin or IGF-1 activates several downstream signal transduction pathways, including AKT and mitogen-activating protein kinase (MAPK). These pathways are thought to coordinate adipogenic effects^6^. Because insulin receptor is not expressed in the early stage of adipogenesis, insulin is thought to function through IGF-1 receptor^11^. On the other hand, other signaling mechanisms, such as that of dexamethasone, and their roles in the process of adipogenesis are not fully elucidated to date.

Dexamethasone, a synthetic glucocorticoid, promotes differentiation of cultured preadipose cell lines and primary adipocytes. This effect is believed to be mediated by the glucocorticoid receptor (GR), which belongs to nuclear receptor superfamily^12^. However, the transcriptional targets and mechanism of glucocorticoid involvement in the adipose differentiation program is unclear. C/EBPδ is reported as one of the targets of glucocorticoids in 3T3-L1 cells^13^,^14^, but C/EBPδ-overexpressing cells still require dexamethasone treatment, indicating the presence of other critical factor(s) for activity^7^. Also, dexamethasone was reported to down-regulate the expression of preadipocyte factor-1 (pref-1), a well-known suppressor of adipogenesis^15^, and with this repression of pref-1, the glucocorticoid signaling is thought to play a role in priming preadipocytes for the commitment to adipogenesis^16^. Recently, an osteogenic factor Runx2, a member of the runt-related transcription factor family, was down-regulated after dexamethasone treatment in 3T3-L1 cells^17^. Although these studies partially explain the effect of glucocorticoids, namely dexamethasone, on the adipogenic differentiation program, we currently do not know whether critical regulators are directly regulated by glucocorticoids, participating in a complex signaling pathway during adipogenesis.

Dexras1 (also known as Rasd1), a Ras family small G protein, was discovered because its expression is markedly induced by dexamethasone treatment^18^. Dexras1 functions as a signal transducer of multiple signaling pathways, including iron homeostasis, growth hormone secretion, and circadian rhythm^19^,^20^. Recently, we reported that Dexras1 is essential for the adipogenic differentiation program, as adiposity and high-fat-diet-induced weight gain are reduced in Dexras1 knockout mice^21^. However, the exact mechanism of Dexras1-mediated adipogenesis has not been fully characterized. In this report, we elucidated the mechanism by which Dexras1 mediates adipogenesis, revealing that glucocorticoid signaling is linked to IGF-1 signaling by means of Dexras1. After Dexras1 is expressed by glucocorticoids, insulin/IGF-1 treatment causes a translocation of Dexras1 to the plasma membrane. Consequently, by participating in IGF-1 signaling, Dexras1 leads to an activation of MAPK and C/EBPβ, which induces the adipogenic program.

## Results

### Dexras1 is essential for MAPK activation and C/EBPβ phosphorylation

**Figure 1:**
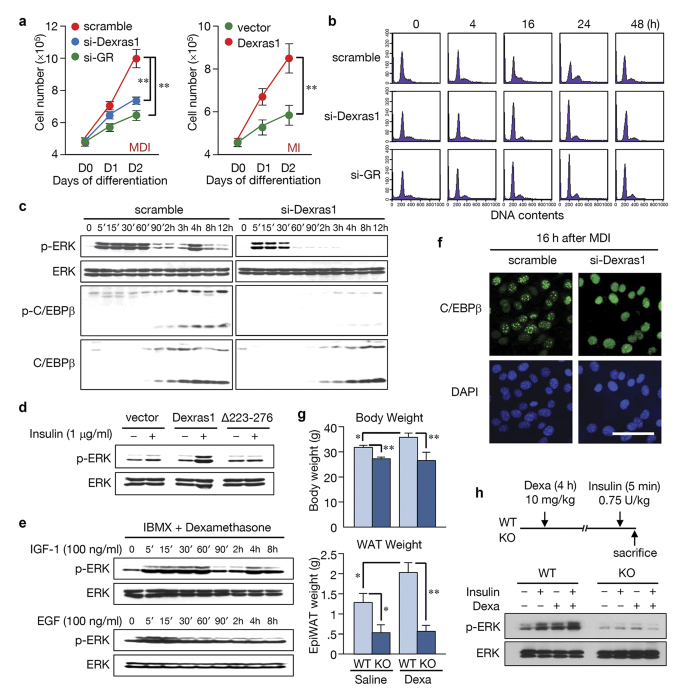
Dexras1 is required for mitotic clonal expansion and activates ERK during adipogenesis. (**a–d**) 3T3-L1 cells were transfected with scramble, si-Dexras1, or si-GR, and then differentiated using IBMX, insulin, and dexamethasone (MDI). Alternatively, 3T3-L1 cells were transfected with pcDNA3 vector or pcDNA3-Dexras1-FLAG and differentiated by IBMX and insulin (MI), as indicated. (**a**) Cell proliferation determined by cell counting at day 1 and 2 after induction of differentiation. Data are presented as the mean ± s.d. **P < 0.01. (**b**) DNA contents were analyzed by flow cytometry at the indicated time points. (**c**) The phosphorylated ERK and C/EBPβ were detected by western blot at indicated time points. (**d**) 3T3-L1 cells transfected with indicated overexpression vectors were treated with 1 μg/ml insulin. Cell lysates were analyzed after 4 h. Schematic drawing of wild type and mutant of Dexras1 used in this study is shown in Supplementary Fig. S3. (**e**) 3T3-L1 cells were stimulated either with IGF-1 (100 ng/ml) or EGF (100 ng/ml) in the presence of IBMX and dexamethasone. (**f**) 3T3-L1 preadipocytes were induced to differentiate with the standard protocol with either scramble or si-Dexras1. Cells were fixed at indicated times and subjected to immunofluorescence analysis with antibody against C/EBPβ and 4′,6-diamidino-2-phenylindole. Fluorescence images were obtained by confocal microscopy. Scale bar = 50 μm. (**g**) Wild type and Dexras1 knockout (KO) mice were subjected to dexamethasone treatment (10 mg/kg) for 5 weeks, three times a week, by intraperitoneal (IP) injection. These mice were fed with high fat diet to maximize the dexamethasone-induced weight gain. Body weights and weights of epididymal fat pads were measured. Data are presented as the mean ± s.d. *P < 0.05, **P < 0.01. The *P* values of body weights were 0.017 (WT-S vs. WT-D), 0.003 (WT-S vs. KO-S), 0.007 (WT-D vs. KO-D), and 0.803 (KO-S vs. KO-D), and the P values of epididymal fat pads were 0.015 (WT-S vs. WT-D), 0.021 (WT-S vs. KO-S), 0.001 (WT-D vs. KO-D), and 0.805 (KO-S vs. KO-D), respectively. (**h**) Wild type and Dexras1 KO mice were injected with dexamethasone and insulin as shown. Epididymal fat pads were homogenized and western blot was performed.

Earlier, we reported that Dexras1 is required for adipogenic differentiation, but the underlying molecular mechanisms have not been characterized^21^. The mitotic clonal expansion of 3T3-L1 cells involves the sequential activation of C/EBPβ protein by MAPK and glycogen synthase kinase 3β (GSK3β). These regulators are required for the expression of the two principal adipogenic factors, C/EBPα and peroxisome proliferator-activated receptor γ (PPARγ)^14^,^22^,^23^,^24^. To elucidate how Dexras1 is involved in adipogenesis, we assessed whether Dexras1 is associated with mitotic clonal expansion. In agreement with are previous results, 3T3-L1 cells were not differentiated in the absence of Dexras1 or glucocorticoid receptor (GR) (Supplementary Fig. S1). Further, the overexpression of Dexras1 resulted in adipogenesis even in the absence of dexamethasone (Supplementary Fig. S1). These results correlated with the expression of adipocyte-specific genes, C/EBPα and PPARγ (Supplementary Fig. S1). Notably, the cell cycle inhibitors p27 and p21 were maintained at high level with si-Dexras1 or si-GR treatment, while Dexras1 overexpression with IBMX and insulin (MI) resulted in the suppression of p27 and p21 expression (Supplementary Fig. S1). Consistently, cell numbers during the differentiation with si-Dexras1/si-GR or the overexpression of Dexras1 demonstrated that mitotic clonal expansion is dependent on the presence of Dexras1 (Fig. 1a). These results were confirmed by FACS analysis, indicating that Dexras1 is required for the G1-S transition of the cell cycle (Fig. 1b; Supplementary Fig. S1). These results suggest that Dexras1 signaling is essential for clonal expansion during adipogenesis.

Because Dexras1 has a Ras-like domain^21^, we hypothesized that Dexras1 is associated with MAPK activation pathway, which is activated by classical Ras proteins in many cell types. In 3T3-L1 cells, MAPK activation is required for early phase of differentiation, but prolonged activation of MAPK suppresses terminal differentiation^5^. We found that suppression of Dexras1 expression by siRNA abolished pERK at ~4 h after initiating differentiation in 3T3-L1 cells (Fig. 1c). This time point is when C/EBPβ protein appears in the cells, and C/EBPβ should be phosphorylated by pERK for its activation^24^. Although the immediate pERK (5 to 30 min) was not completely diminished by si-Dexras1, the C/EBPβ protein was not phosphorylated without the second activation of ERK at ~4 h (Fig. 1c; Supplementary Fig. S2). This phenomenon was also observed in mouse embryonic fibroblast (MEF) cells of Dexras1 KO mice (Supplementary Fig. S2), suggesting that Dexras1 is involved in the serial events of adipogenesis such as MAPK activation, C/EBPβ phosphorylation, and mitotic clonal expansion.

Notably, other insulin/IGF-1 signaling molecules, such as p85, AKT, or mTOR were not altered by si-Dexras1 (Supplementary Fig. S1). Previously, we have shown that a C-terminal domain of 52 amino acids in Dexras1 (Supplementary Fig. S3) is critical for activity^21^. To confirm this finding, 3T3-L1 cells with the transient overexpression of full-length Dexras1 or C-terminal deletion mutant (amino acids 223–276) were tested. Wild type Dexras1 expression led to adipocyte differentiation (Supplementary Fig. S3) and ERK activation in the presence of insulin, while the C-terminal deletion mutant did not (Fig. 1d). These data further demonstrate the importance of this C-terminal portion of the protein to Dexras1 function.

Dexras1 is known to participate in the effects of nNOS-CAPON association in the brain, negatively regulating the phosphorylation of ERK^25^,^26^. Our results showed that Dexras1 activates ERK in 3T3-L1 cells. Therefore, we hypothesized that this discrepancy may be due to a difference in cell type or extracellular signaling. To explore this possibility, we tested the effect of different growth factors; IGF-1 and epidermal growth factor (EGF), on ERK activation in 3T3-L1 cells. Although both signals induced the immediate phosphorylation of ERK (~15 min), the activation of ERK at 4 h appeared to be unique in IGF-1 (Fig. 1e; Supplementary Fig. S4) or insulin signaling (Fig. 1c). Furthermore, ERK activation at 4 h was observed in 3T3-L1 and NIH3T3 cells, but not in 293T cells, suggesting that the effect of Dexras1 depends on the cell type (Supplementary Fig. S4). ERK phosphorylation in response to insulin was not observed in MEF cells from Dexras1 knockout mice, but reappeared after ectopic expression of Dexras1 (Supplementary Fig. S4). The failure of ERK-dependent phosphorylation of C/EBPβ by si-Dexras1 abolished a characteristic “punctate” pattern in immunofluorescent staining (Fig. 1f; time-course results shown in Supplementary Fig. S5), which is a hallmark of the acquisition of DNA binding activity of C/EBPβ with its centromeric localization during S phase (~16 h) of mitotic clonal expansion^27^.

In order to address whether Dexras1-dependent ERK activation happens *in vivo*, we used wild type and Dexras1 knockout mice whether they respond to dexamethasone. Intraperitoneal injection of dexamethasone caused the dramatic elevation of Dexras1 expression in white adipose tissue of wild type mice, but not in that of knockout mice (Supplementary Fig. S6). Next, mice were subjected to intraperitoneal injection with saline or dexamethasone for 5 weeks, three times a week. These mice were fed with high fat diet, which we found to be an effective way to observe dexamethasone-induced weight gain. As a result, we found that the body weight and epididymal fat pads were significantly lower in Dexras1 knockout mice, compared to wild type mice, after the dexamethasone treatment (Fig. 1g), strongly suggesting that Dexras1 is critical for glucocorticoid response in adipose tissue. More importantly, we tested whether Dexras1 mediates the MAPK signaling *in vivo*. We treated dexamethasone in wild type and Dexras1 knockout mice, and after 4 h (at which Dexras1 is induced in adipose tissue) we injected insulin for 5 min and then sacrificed the mice. As a result, ERK was activated in white adipose tissue of wild type mice in response to dexamethasone and insulin, but not in that of Dexras1 knockout mice (Fig. 1h). This *in vivo* result further provides a direct evidence for the role of Dexras1 in ERK activation. Taken together, we concluded that glucocorticoid signaling of expressing Dexras1 is linked to IGF-1 signaling, leading to MAPK and C/EBPβ phosphorylation during adipogenesis.

### Dexras1 translocates to the plasma membrane upon insulin signaling.

**Figure 2:**
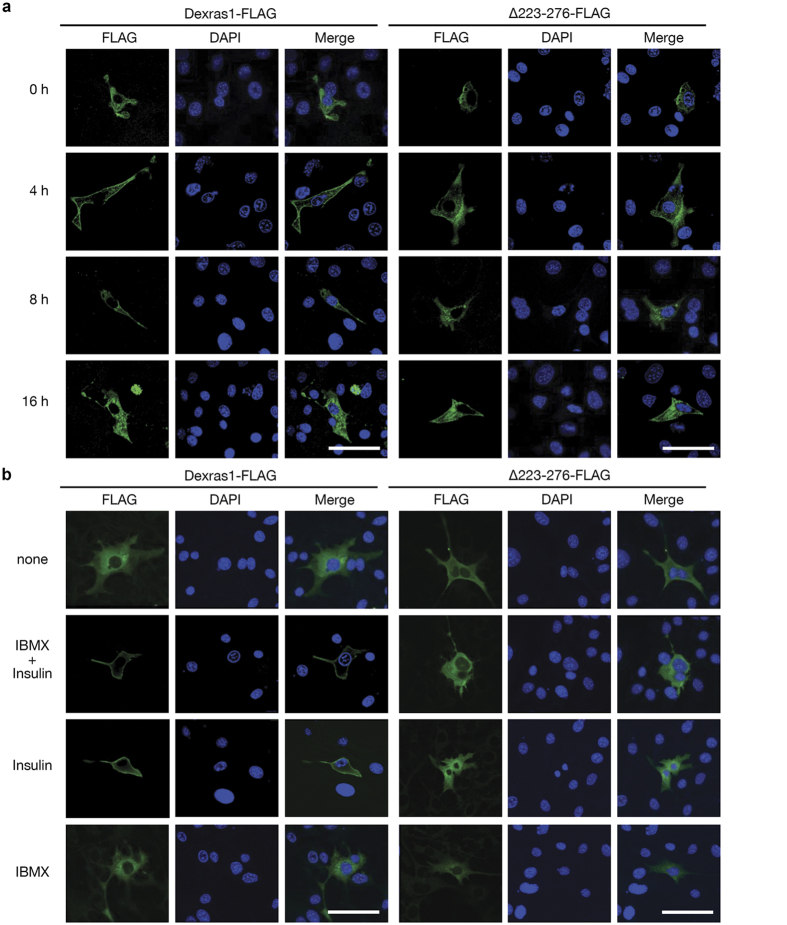
Dexras1 translocates to the plasma membrane in response to insulin signaling. 3T3-L1 cells were transfected with pcDNA3-Dexras1-FLAG or pcDNA3-∆223-276-FLAG vector. Cellular localization of Dexras1 was analyzed by confocal microscopy. Transfected cells were incubated with (**a**) IBMX, dexamethasone, and insulin for the times indicated, or (**b**) various differentiation cocktails for 1 h. To visualize Dexras1 protein, cells were fixed and subjected to immunofluorescence analysis with antibody against FLAG (green). Nuclei were stained with DAPI and fluorescence was visualized by confocal microscopy. Scale bar = 50 μm.

Our results indicate that insulin/IGF-1, but not IBMX, induces the activation of ERK in the presence of Dexras1. These data imply that glucocorticoid-induced Dexras1 is associated with insulin or IGF-1 signaling, at least for the purpose of ERK activation. Notably, the overexpression of Dexras1 alone did not activate ERK until insulin was added (Fig. 1d). We hypothesized that some features or properties of Dexras1 should be changed by insulin or IGF-1 treatment. Therefore, we analyzed the cellular localization of Dexras1 by confocal microscopy. 3T3-L1 cells expressing Dexras1-FLAG were treated with a hormone cocktail (IBMX and insulin, designated as MI) for differentiation induction. Before the addition of the hormone cocktail (0 h), Dexras1-FLAG was observed throughout the cytoplasm. However, Dexras1-FLAG translocated to the membrane after 4 h of MI treatment and remained at the membrane at 8 h (Fig. 2a, left). The deletion of C-terminal amino acid 223–276, which cannot induce adipogenesis or MAPK activation (Fig. 1d; Supplementary Fig. S3) did not translocate to the membrane (Fig. 2a, right). The membrane localization of Dexras1 was clearly due to the insulin signaling, as shown in Fig. 2b. These data suggest that Dexras1 translocation is accomplished through C-terminal domain. Taken together, these data demonstrate that Dexras1 is a mediator of cross-talk between glucocorticoid and insulin (or IGF-1) signaling in adipogenesis.

### Dexras1 mediates adipogenesis by the IGF-1 pathway

**Figure 3:**
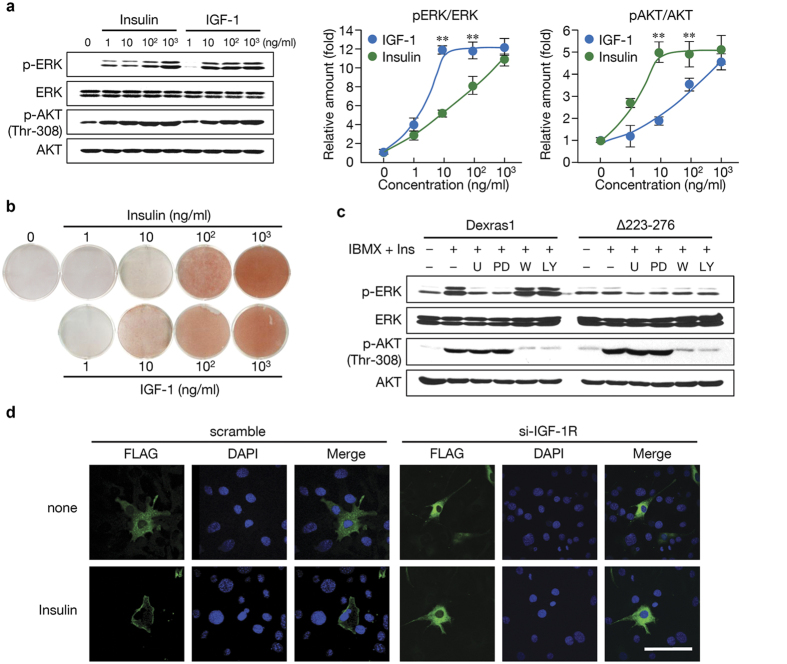
Dexras1 is required for IGF-1-induced ERK activation, but not for AKT activation, in adipogenesis. (**a**) pcDNA3-Dexras1-FLAG overexpressing 3T3-L1 cells were incubated with various concentrations of insulin or IGF-1 for 30 min. Immunoblots are a representative image of three independent experiments, and the activation levels of ERK or AKT were measured and plotted. Data are presented as the mean ± s.d. **denotes *P* < 0.01. (**b**) 3T3-L1 cells transfected with pcDNA3-Dexras1-FLAG were differentiated with various concentrations of insulin or IGF-1 in the presence of IBMX. Oil-red-O staining was performed on day 8. (**c**) Cells were transfected with pcDNA3-Dexras1-FLAG or pcDNA3-∆223-276-FLAG. After 30 min of pretreatment with the indicated reagents (U, U0126; PD, PD98059; W, wortmannin; or LY, LY294002) cells were incubated with IBMX and insulin (1 μg/ml) for 10 min. ERK and AKT activation was evaluated by immunoblot analysis. (**d**) Dexras1-overexpressing 3T3-L1 cells were transfected either with scramble or IGF-1R siRNAs, and then stimulated by insulin (1 μg/ml) for 1 h. Cells were fixed and subjected to immunofluorescence analysis with antibody against FLAG (green). Nuclei were stained with DAPI and fluorescence was visualized by confocal microscopy. Scale bar = 50 μm.

Because Dexras1 affected activation of MAPK, but not AKT (Supplementary Fig. S2), we investigated how Dexras1 is involved in insulin and IGF-1 pathways. Despite numerous studies on differences between insulin and IGF-1 pathways, these pathways are virtually indistinguishable *in vitro*, presumably due to the supra-physiological concentration of these molecules. We exposed 3T3-L1 cells overexpressing Dexras1 to a range of insulin or IGF-1 concentrations. As shown in Fig. 3a, insulin caused gradual increase of pERK with increasing dose up to 1,000 ng/ml, whereas AKT was fully activated by even a low concentration of insulin (10 ng/ml). In contrast, IGF-1 showed the inverse pattern: ERK activation was observed with relatively low concentration of IGF-1 (10 ng/ml); at which AKT phosphorylation (Thr-308) did not reach the maximum. This result indicates that, at physiological concentrations, insulin may prefer AKT signaling, while IGF-1 signaling is mainly associated with MAPK pathway. Consistently, low dose (10 ng/ml) of IGF-1, but not insulin, could induce the differentiation (Fig. 3b), suggesting that the early activation of ERK to a proper level is critical to the adipogenesis.

Insulin/IGF-1 signaling involves two main pathways, one via phosphatidylinositol 3-kinase (PI3K) and the other via MAPK^28^. To further define a role of Dexras1 in the insulin/IGF-1 signaling, we tested whether inhibitors of these signaling pathways affected their dependency on Dexras1. The MAPK (ERK) activation in 3T3-L1 cells overexpressing Dexras1 was almost completely abolished by the addition of U0126 or PD98059 compound, which are known inhibitors of MAPK pathway (Fig. 3c, left). In contrast, PI3K inhibitors, wortmannin or LY294002 compound, did not affect Dexras1-involved activation of MAPK. Consistent with the results in a deletion mutant of Dexras1 (Fig. 3c, right), these data suggest that Dexras1 is only involved in the ERK pathway, as AKT signaling remains intact regardless of the presence of functional Dexras1. This result is also observed in MEFs from Dexras1 KO mice (Supplementary Fig. S7). Furthermore, in the absence of IGF-1 receptor (IGF-1R), insulin did not affect either the translocation of Dexras1 to the plasma membrane (Fig. 3d) or Dexras1-involved activation of MAPK (Supplementary Fig. S7), indicating that Dexras1 activates MAPK primarily through IGF-1R.

**Figure 4:**
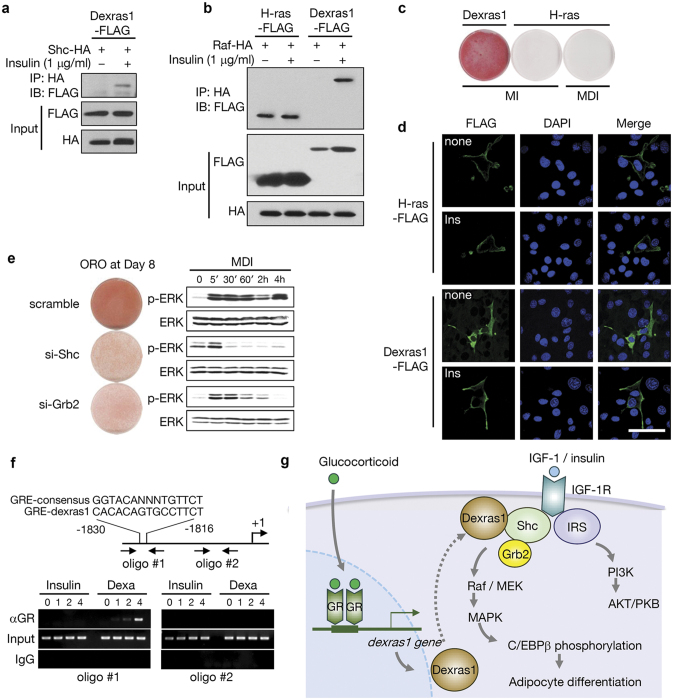
Dexras1-induced activation of MAPK is dependent on Shc-Grb2 pathway. (**a**) Immunoprecipitation and western blotting of Dexras1-FLAG and Shc-HA in 3T3-L1 cells. After stimulation of insulin (1 μg/ml) for 1 h, cell lysates were immunoprecipitated with anti- HA antibody and analyzed with anti-FLAG antibody. (**b**) Immunoprecipitation and western blotting of Dexras1-FLAG or H-ras-FLAG and Raf-HA. After stimulation of insulin (1 μg/ml) for 1 h, cell lysates were Immunoprecipitated with anti-HA antibody and then analyzed with anti-FLAG antibody. (**c**) 3T3-L1 cells were transfected with pcDNA3-Dexras1-FLAG or pcDNA3-H-ras-FLAG, and then differentiation was induced using the indicated hormone cocktails (MI, IBMX and insulin; MDI, IBMX, dexamethasone, and insulin). Oil-red-O staining was carried out on day 8. (**d**) 3T3-L1 cells were transfected with pcDNA3-Dexras1-FLAG or pcDNA3-H-ras-FLAG and incubated with insulin (ins) for 1 h. Cells were fixed and subjected to immunofluorescence analysis with antibody against FLAG (green). Nuclei were stained with DAPI and fluorescence was visualized by confocal microscopy. Scale bar = 50 μm. (**e**) 3T3-L1 cells were transfected with control, Shc, or Grb2 siRNAs, and differentiation was induced by MDI. Oil-red-O (ORO) staining was performed on day 8. ERK activation was evaluated by immunoblot at the indicated time points. (**f**) A chromatin immunoprecipitation study. The promoter region of Dexras1 was presented with putative GRE, oligonucleotides for PCR, and transcription start site (+1). Anti-GR antibody (αGR) was used for this assay. The resulting PCR products were resolved in 1.2% agarose gel. (**g**) A schematic model of Dexras1 functions in adipogenesis. Dexras1 is expressed by glucocorticoid and its receptor complex, remaining in the cytoplasm until insulin or IGF-1 is treated. When IGF-1R is activated by the hormone, Dexras1 translocates to the plasma membrane interacting with Shc, thereby transferring the IGF-1 signaling through Shc/Grb2, Raf, MEK, and MAPK. The Dexras1 is involved only in MAPK pathway, not interfering AKT/PKB signal. The activated MAPK then phosphorylates C/EBPβ protein, which mediates mitotic clonal expansion and further adipogenic program.

How is Dexras1-dependent activation of MAPK accomplished? Earlier reports suggest that IGF-1 pathway is involved in adipogenesis through Shc-mediated activation of MAPK^29^,^30^. We asked whether Dexras1 constitutes a complex of IGF-1R signaling by binding to Shc and/or Grb2 in 3T3-L1 cells. To clarify this signaling system, we sought to identify the binding partners of Dexras1. Using immunoprecipitation techniques, we found that Dexras1-FLAG could interact with HA-Shc upon insulin treatment (Fig. 4a). Similarly, we also detected an interaction between Dexras1-FLAG and HA-Raf in the presence of insulin (Fig. 4b). By contrast, H-ras interacted with Raf regardless of insulin treatment (Fig. 4b), and could not differentiate the cells even in the full differentiation media (Fig. 4c). This is probably because sustained activation of MAPK rather suppresses differentiation^5^. Consistently, H-ras localized to the plasma membrane independently of insulin treatment (Fig. 4d), suggesting that Dexras1 is selectively involved in the regulation of IGF-1 signaling. To confirm the involvement of Dexras1 in IGF-1 signaling, Dexras1-overexpressing 3T3-L1 cells were introduced to siRNA-mediated suppression of Shc or Grb2. As shown in Fig. 4e, Dexras1-induced activation of MAPK was largely dependent on Shc or Grb2 expression.

To make sure that Dexras1 is a direct transcriptional target of the glucocorticoid receptor, we performed the chromatic immunopreciptiation assay (ChIP). Computer analysis revealed a putative glucocorticoid response element (GRE) in the region of -1,830 to -1816 from the transcription start site, and ChIP assay showed a clear binding of glucocorticoid receptor to this region (Fig. 4f). Taken together, we suggest that the glucocorticoid and IGF-1 signaling in 3T3-L1 cells involves serial cellular events; glucocorticoids directly induce the expression of Dexras1, which translocates to the membrane (probably recruited by Shc) in response to the activation of IGF-1/IGF-1 receptor complex. The signal is transferred through Shc/Grb2, Dexras1, and Raf-MEK-MAPK, which is independent of PI3K-AKT signaling. This model is summarized in Fig. 4g.

## Discussion

One of the long standing questions in adipogenesis is the role of glucocorticoids. Do these compounds act only to promote the differentiation process, or are they absolutely required for optimal differentiation? Because a number of studies using various origins of preadipocytes have showed different answers to this question, the answer cannot be easily concluded. In serum-free medium, for example, porcine, rabbit, and human preadipocytes exhibited a strict glucocorticoid dependency for optimal differentiation; whereas rat preadipocytes differentiated extensively without glucocorticoids^31^. In 3T3-L1 adipogenesis, however, dexamethasone treatment was required for optimal differentiation, even in cells transfected to overexpress C/EBPα or PPARγ. Thus, to provide mechanistic insight into the role of dexamethasone in adipogenesis, we sought to identify a critical mediator of glucocorticoid signaling during the adipogenesis process. Our findings establish a major adipogenic role for Dexras1 that it participates in the right-timing activation of MAPK, thereby phosphorylating C/EBPβ protein for the progression of mitotic clonal expansion as well as for the expression of adipocyte-specific regulators C/EBPα or PPARγ.

Our results provide several important advances in understanding the mechanism of adipogenesis. First, we identified a critical downstream effector of glucocorticoid action in early adipogenesis. Since 3T3-L1 cell line was introduced in 1974 as its differentiation into lipid-laden fat cells^9^, many hormonal factors and their signaling pathways, possessing pro- or anti-adipogenic effects, have been studied including the factors involved in preadipocyte commitment or terminal differentiation program. Of these, glucocorticoid signaling has not been thoroughly studied so far, partially due to the fact that target genes of activated glucocorticoid receptor in the adipogenic program were poorly identified. Our data show that Dexras1 expression can substitute for dexamethasone treatment in the differentiation protocol, making it clear that the requirement of glucocorticoids in adipogenesis can be explained by the existence of Dexras1. Thus, glucocorticoid signaling through Dexras1 in these fat cells might be important for adipocyte proliferation, both in over-nutrition and in glucocorticoid excess status.

Second, our data provide the details of insulin or IGF-1 signaling in adipogenesis. Dexras1 translocates to the membrane through the C-terminal domain (Fig. 2) and in the absence of Dexras1, MAPK or MAPK-directed C/EBPβ activation was not accomplished, even in the full differentiation medium, indicating that insulin/IGF-1 signaling to MAPK is clearly associated with Dexras1. IGF-1R-mediated signaling, but not insulin receptor, is important for early adipogenesis^11^. Our results also show that IGF-1 is more competent for Dexras1-driven MAPK activation than is insulin. Signaling by IGF-1 in adipocyte differentiation is complex, primarily involving the MAPK pathway. Because of its importance in adipocyte mitogenic activity, IGF-1 signaling has been extensively studied using 3T3-L1 cells, establishing the involvement of Shc as an adapting molecule^30^. This finding is strengthened by our results, which also demonstrated the involvement of Dexras1. It is very important that IGF-1 signaling should be decreased once the mitotic clonal expansion is initiated. In this regard, it is interesting to note that the ectopic expression of activated Ras induced adipocyte differentiation^32^, but persistent MAPK activation inhibits adipogenesis, either by through phosphorylation of PPARγ^33^ or by other mechanisms^5^. Therefore, the timing of MAPK activation is critical for early adipogenesis. Dexras1 was expressed at ~4 h of differentiation induction, mediating the “right-timing” phosphorylation of C/EBPβ, but then disappeared, preventing surplus activation of MAPK. It is also interesting to investigate whether Dexras1 participates in a commitment state during adipogenesis, because the glucocorticoid treatment reduces pref-1, thereby inducing a cell fate determinant in preadipocytes^16^. This is highly possible because pref-1 is not down-regulated in the absence of Dexras1^21^.

Glucocorticoid treatment elicits Cushing’s syndrome with increased visceral adiposity, which might be mediated by Dexras1. The other functions of glucocorticoids may involve Dexras1 as well, and is a subject for future research. In order to provide a mechanism by which we can reduce side effects of glucocorticoids, it is also important to investigate whether Dexras1 is involved in anti-inflammatory effects of glucocorticoids. In this regard, our report that glucocorticoid-mediated NF-κB suppression is preserved in the absence of Dexras1^34^ might be interesting to note, thus the inhibition of Dexras1 would be a great target for reducing steroid-mediated side effects. Additional future studies should seek to determine the glucocorticoid-mediated effect of adipogenesis *in vivo*, perhaps by using animals treated with an excess glucocorticoids.

## Materials and Methods

### Cell culture and *in vitro* differentiation

The 3T3-L1 and 3T3-F442A preadipocytes were cultured and differentiated into adipocytes as described previously^35^. Briefly, preadipocyte 3T3-L1 cells were maintained in Dulbecco’s modified Eagle’s medium (DMEM) containing 100 U/ml penicillin, 100 μg/ml streptomycin, and 8 μg/ml biotin, supplemented with 10% heat-inactivated calf serum at 37 °C, in an atmosphere of 90% air and 10% CO_2_. To induce differentiation, 2-day post-confluent 3T3-L1 cells (designated day 0) were incubated in DMEM containing 10% FBS, 0.5 mM 3-isobutyl-1-methylxanthine, 1 μM dexamethasone, and 1 μg/ml insulin for 2 days. Cells were then cultured in DMEM containing 10% FBS and insulin for another 2 days, after which they were grown in DMEM containing 10% FBS. Lipid accumulation in the cells was detected by oil-red-O staining on day 8.

### Experimental animals

Six-week-old male C57BL/6J wild type and Dexras1 knockout mice were housed maximum four per cage under 12-h light 12-h dark cycle. For dexamethasone treatment, mice were injected intraperitoneally either normal saline or dexamethasone sodium phosphate (10 mg/kg, Santa Cruz Biotechnology) every 2 days for 5 weeks upon a high fat diet. Animal protocols were performed in accordance with National Institutes of Health guidelines and approved by the Johns Hopkins University Committee on Animal Care and the Committee on Animal Investigations of Yonsei University.

### MEF adipogenesis assays

Wild-type and knockout E14 embryos were isolated from a single heterozygous female that had been paired with a heterozygous male. MEFs were seeded in six-well plates and propagated to confluence. Forty-eight hours later, differentiation was initiated using DMEM containing 10% FBS, 0.5 mM 3-isobutyl-1-methylxanthine, 1 μM dexamethasone, 10 μg/ml insulin and 10 μM rosiglitazone for 2 days. Subsequently, cells were maintained in DMEM supplemented with 10% FBS, 10 μg/ml insulin, and 1 μM rosiglitazone. After 48 h, the medium was replaced with maintenance medium composed of DMEM supplemented with 10% FBS.

### Transient transfection assay

Cells were transiently transfected using microliter volume electroporation of 3T3-L1 preadipocytes with OneDrop MicroPorator MP-100 (Digital Bio) to maximize the transfection efficiency. The cells were trypsinized, washed with 1× PBS, and resuspended in 10 μl of resuspension buffer R with 0.5 μg of plasmid at a concentration of 200,000 cells per pipette. The cells were then microporated at 1,300 V, with two consecutive 20-ms pulses. Following microporation, the cells were seeded in 35-mm cell culture dishes and placed at 37 °C in a 10% CO_2_-humidified atmosphere.

### Small interfering RNA (siRNA)

3T3-L1 cells were plated into 60-mm-diameter dishes 18–24 h prior to transfection. Cells were transfected with control or gene-specific siRNA at 50 nM (Dharmacon) in OPTI-MEM medium using Lipofectamine RNAiMAX (Invitrogen), according to the manufacturer’s protocol. The next day, the medium was replaced with fresh DMEM containing 10% calf serum and the cells were incubated for 24 h before the induction of differentiation. Cellular total RNA and protein were assayed at the indicated time points respectively by RT-PCR and immunoblot. Oil-red-O staining of Dexras1 knockdown was performed on day 8. The siRNA sequences were as follows: si-Dexras1, 5′-UCAAA CAGCA GAUCC UAGAU U-3′; si-GR, 5′-GAUCC CCGAA AGCAU UGCAA CCTCA-3′; si-Shc, 5′-CUACU UGGUU CGGUA CAUGG GTT-3′; si-Grb2, 5′-CAUGU UUCCC CGCAA UUAUT T-3′; si-IGF-1R, 5′-CUUCA GCUGC UGAUA GUCGT T-3;.si-H-ras, 5′-GUGUGU UUGCCA UCAACA UU-3.

### Western blot analysis and antibodies

For protein analysis, cells were washed with ice-cold PBS and lysed in a buffer containing 1% SDS and 60 mM Tris-Cl, pH 6.8. The lysate was mixed briefly using a Vortex, boiled for 10 min, and centrifuged at 12,000 g for 10 min at 4 °C. Protein concentrations were assessed using the BCA assay kit (Pierce). Protein samples of equal amount were separated by SDS-PAGE and transferred to nitrocellulose membranes. Immunoblot analyses were performed using the following antibodies: polyclonal antibody against C/EBPα, polyclonal antibody against C/EBPβ, mouse monoclonal antibody against PPARγ, IGF-1R, pp85, p85, pmTOR, mTPR, pGSK3β, GSK3β and β-actin (Santa Cruz Biotechnology), FLAG (Sigma), HA (Roche), pC/EBPβ, pERK, ERK, pAKT (Thr-308), and AKT antibodies (Cell signaling).

### Gene expression analysis

The level of gene expression was measured by RT-PCR using total RNA from 3T3-L1 cells. Total RNA was isolated from cells or tissues using TRIzol (Invitrogen) according to the manufacturer’s instructions. For quantitative RT-PCR, cDNA was synthesized from 5 μg of total RNA using random hexamer primers and SuperScript reverse transcriptase II (Invitrogen). PCR was performed using the following primers: Dexras1, 5′-CGCCT CTCTA TCCTC ACAGG-3′, 5′-GGTCC AAGCT GCTGT TCTTC-3′; Shc, 5′-CCAAC CATCA CATGC AATCT-3′, 5′-TGACA GGATG GCTGG CTTTG-3′; Grb2, 5′-CAGAA CTGGT ATAAG GCAGA-3′, 5′-GCTCC GCGAC GGAGC CGGGA-3′; GAPDH, 5′-ACCAC AGTCC ATGCC ATCAC-3′, 5′-TCCAC CACCC TGTTG CTGTA-3′.

### Cell count assay

3T3-L1 cells were seeded at a density of 1 × 10^4^ cells/well into 24-well plates, incubated for 24 h and synchronized to quiescence by serum starvation for 12 h. At indicated time points, the cells were trypsinized and cell numbers were determined using an automated cell counter, ADAM (NanoEnTek), according to the manufacturer’s instructions.

### Cell cycle analysis

3T3-L1 cells seeded into 100-mm culture dish were incubated for 24 h and were synchronized to quiescence by serum starvation for 12 h. The cells were then trypsinized and collected by centrifugation at 1,000 rpm for 5 min. The cell pellets were fixed in 70% ethanol at −70 °C for 1 h, and then they washed twice with ice-cold PBS and resuspended in propidium iodide staining solution (5 μg/ml propidium iodide, 0.7 μg/ml ribonuclease A, 10 mM Tris-Cl, pH 7.0, 1 mM NaCl and 0.1% NP-40). Following the incubation in the dark for 30 min at room temperature, cellular DNA was measured based on the propidium iodide signal of the cells, and cell cycle profiles were determined using a FACSCalibur™ (Becton Dickinson Biosciences) and CellQuest Pro™ software.

### Immunocytochemistry

3T3-L1 cells were transfected with pcDNA3-Dexras1-FLAG, deletion constructs, or H-ras, and then they were differentiated on coverslips in 6-well plates as described above. At the indicated times, cells were washed with PBS, fixed in 4% formaldehyde for 10 min, permeabilized with 0.2% Triton X-100 for 20 min on ice, and then blocked in 3% bovine serum albumin in PBS for 1 h. Cells were then incubated in a blocking solution containing FLAG antibody (1:200 dilution) for 1 h, followed by the secondary antibody (anti-mouse IgG-fluorescein isothiocyanate) for 2 h. The cells were mounted in 4′,6-diamidino-2-phenylindole. Cells were then visualized with Confocal Laser Scanning Microscope (Olympus FV1000).

### Immunoprecipitation

For immunoprecipitation experiments, cells were scraped into ice cold lysis buffer (30 mM Tris-Cl, pH 7.5, 150 mM NaCl, 1% Triton X-100, 0.2% sodium deoxycholate, 10 mM NaF, 1 mM Na_3_VO_4_ and 1 mM PMSF) with Complete Protease Inhibitor cocktail (Roche). Cleared extracts incubated with protein A/G agarose (Santa Cruz Biotechnology) and appropriate antibodies for overnight at 4 °C. Immunoprecipitates were washed three times with wash buffer (30 mM Tris-Cl, pH 7.5, 300 mM NaCl, 5 mM NaF and 0.1% Triton X-100), resuspended in 2× sample buffer and boiled for 10 mins. Protein complexes were resolved by SDS-PAGE.

### Immunofluorescent staining

3T3-L1 cells were differentiated on coverslips in 6-well plates and differentiated. At the times indicated, cells were washed with PBS, fixed in 4% formaldehyde for 10 min, permeabilized with 0.2% Triton X-100 for 20 min on ice, and then blocked in 3% bovine serum albumin in PBS for 1 h. Cells were then incubated in blocking solution containing C/EBPβ antibody (1:250 dilution) for 1 h, followed by secondary antibody (anti-rabbit IgG-fluorescein isothiocyanate) for 1 h. The cells were mounted in 4′,6-diamidino-2-phenylindole.

### Chromatin Immunoprecipitation (ChIP) assay

Nuclear proteins were cross-linked to genomic DNA with 1% formaldehyde for 10 min at room temperature. Cells were scraped into ice-cold PBS containing protease inhibitors and following centrifugation, pellets were resuspended in lysis buffer (0.1% SDS, 150 mM NaCl, 1 mM EDTA, 1% Triton X-100, 5 μM leupeptin, 2 μM pepstatin, 1 μM aprotinin, 20 μM PMSF and 50 mM Tris-Cl, pH 7.5) and incubated on ice for 10 min. Lysates were sonicated and centrifuged after which the resulting supernatants were incubated with rotation in the presence of anti-GR antibody (1 μg). Following the addition of protein A/G-agarose (SantaCruz Biotechnology) reactions were incubated for 4 h at 4 °C and then immune complexes were precipitated by centrifugation. After sequential washes with wash buffer (0.1% SDS, 1% Triton X-100, 1 mM EDTA, 500 mM NaCl, 50 mM Tris-Cl, pH 7.5, and 0.1% Na-deoxycholate) and LiCl wash buffer (250 mM LiCl, 0.5% NP-40, 0.1% Na-deoxycholate, 1 mM EDTA and 20 mM Tris-Cl, pH 7.5), the beads were washed twice with TE buffer. Following centrifugation, the resulting immune complexes were resuspended in 200 μl elution buffer (50 mM Tris-Cl, pH 8.0, 1 mM EDTA, 1% SDS and 50 mM NaHCO_3_) and incubated for 10 min at 65 °C. After another centrifugation step, the supernatant was collected and cross-linking reversed by adding NaCl to a final concentration of 0.3 M. The remaining proteins were digested with proteinase K and genomic DNA fragments recovered by phenol-chloroform extraction, followed by ethanol precipitation and resuspension in sterile H_2_O. Mouse genomic sequences containing the putative glucocorticoid receptor binding sites (GRE) and a region further upstream from the Dexras1 promoter (oligo#1; -1882 region, and oligo#2; -659 region, respectively) were amplified using the following primers: oligo#1 set (5′-AGGGG AATGG TGACA TTGGC-3′ and 5′-AGCAT AGAGT GTCAC TGCAG-3′), and oligo#2 set (5′-GTACA GACTC TCCTC TACTG-3′ and 5′-GGACC CGCTG GGATG CCTAA-3′).

### Statistical analysis

All results are expressed as mean ± s.d. Statistical comparisons of groups were made using an unpaired Student’s t test or two-way ANOVA.

## Additional Information

**How to cite this article**: Kim, H. J. *et al*. Dexras1 links glucocorticoids to insulin-like growth factor-1 signaling in adipogenesis. *Sci. Rep.* **6**, 28648; doi: 10.1038/srep28648 (2016).

## Supplementary Material

Supplementary Information
