# Supplementary material for: Dexras1 links glucocorticoids to insulin-like growth factor-1 signaling in adipogenesis
Source: Sci Rep. 2016 Jun 27;6:28648. doi: 10.1038/srep28648 (PMC4921850; doi:10.1038/srep28648)
Supplement: Supplementary Information [file srep28648-s1.pdf]

Supplementary Materials

Dexras1 links glucocorticoids to insulin-like growth factor-1 signaling in adipogenesis

Hyo Jung Kim, Jiyoung Y. Cha, Jo Woon Seok, Yoonjeong Choi, Bo Kyung Yoon, Hyeonjin Choi, Jung Hwan Yu, Su Jin Song, Ara Kim, Hyemin Lee, Daeun Kim, Ji Yoon Han, Jae-woo Kim

Contents : Supplementary Figures and Legends (Figures S1-S7)

Supplementary Figure S1

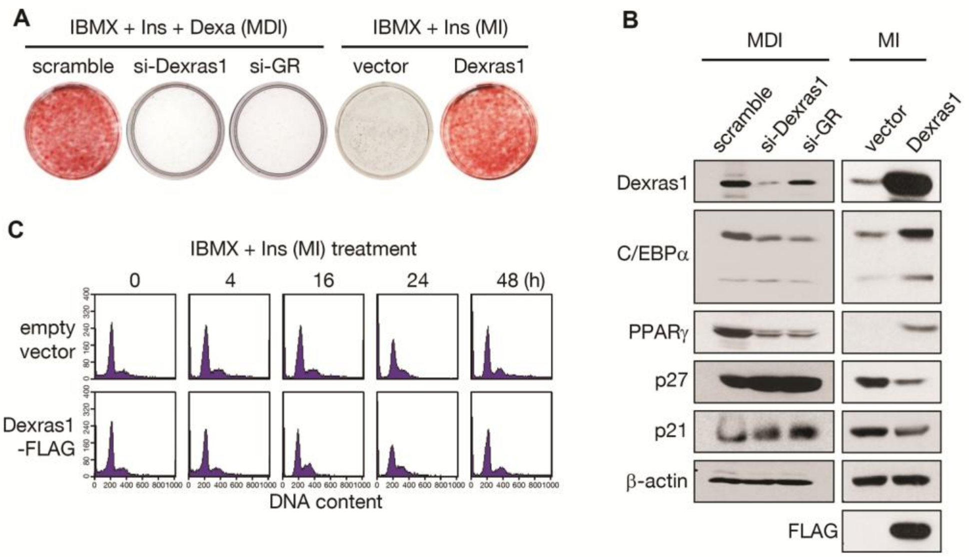

Supplementary Figure S1. Dexras1 is required for mitotic clonal expansion during adipogenesis. 3T3-L1 cells were transfected with scramble, si-Dexras1, or si-GR, and then differentiated using IBMX, insulin, and dexamethasone (MDI). Alternatively, 3T3-L1 cells were transfected with pcDNA3 vector or pcDNA3-Dexras1-FLAG and differentiated by IBMX and insulin (MI), as indicated. (**A**) Oil-red-O staining at day 8 of induction. **(B)** Expression of adipogenic markers and the cell cycle regulator by western blot. **(C)** DNA contents were analyzed by flow cytometry at the indicated time points.

Supplementary Figure S2

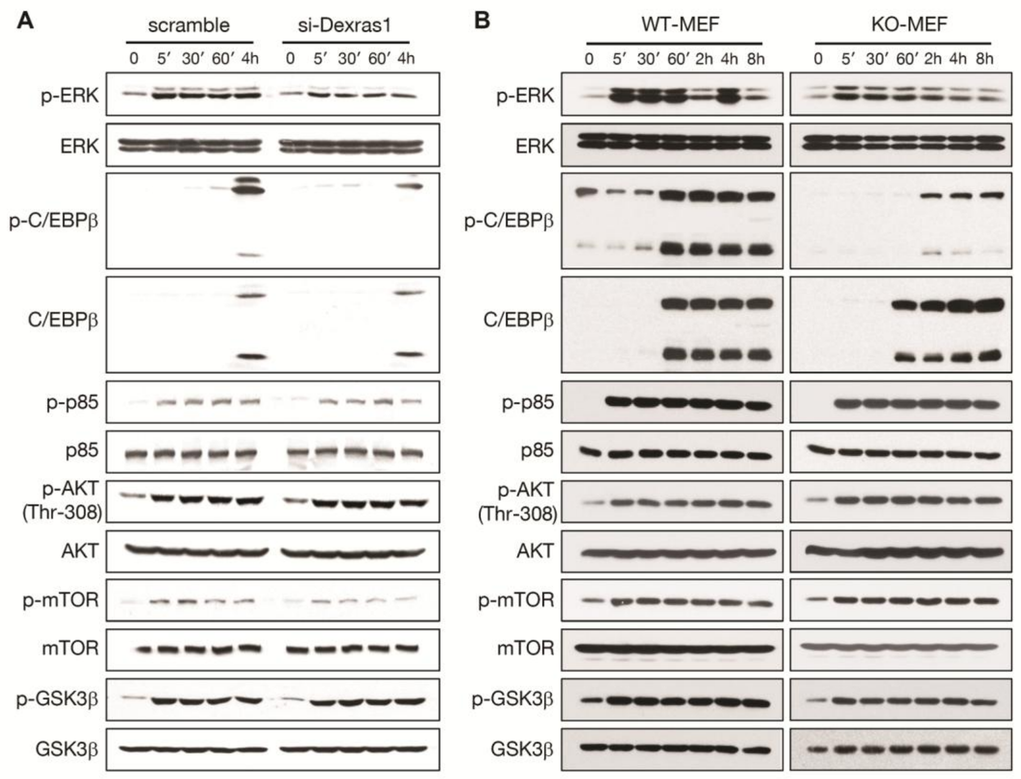

Supplementary Figure S2. Dexras1 regulates the ERK activation, but not PI3K/AKT or other signaling pathway. **(A)** Cells were transfected with Dexras1 siRNA. After 24 h, cells were treated with IBMX, dexamethasone, and insulin, and then cells were harvested at the indicated time points. Effects of Dexras1 were measured by immunoblot analysis using the indicated antibodies. **(B)** MEF cells from wild type or Dexras1 KO mice were prepared and differentiation was induced by IBMX, dexamethasone, insulin, and rosiglitazone. Cell lysates were analyzed by western blot.

Supplementary Figure S3

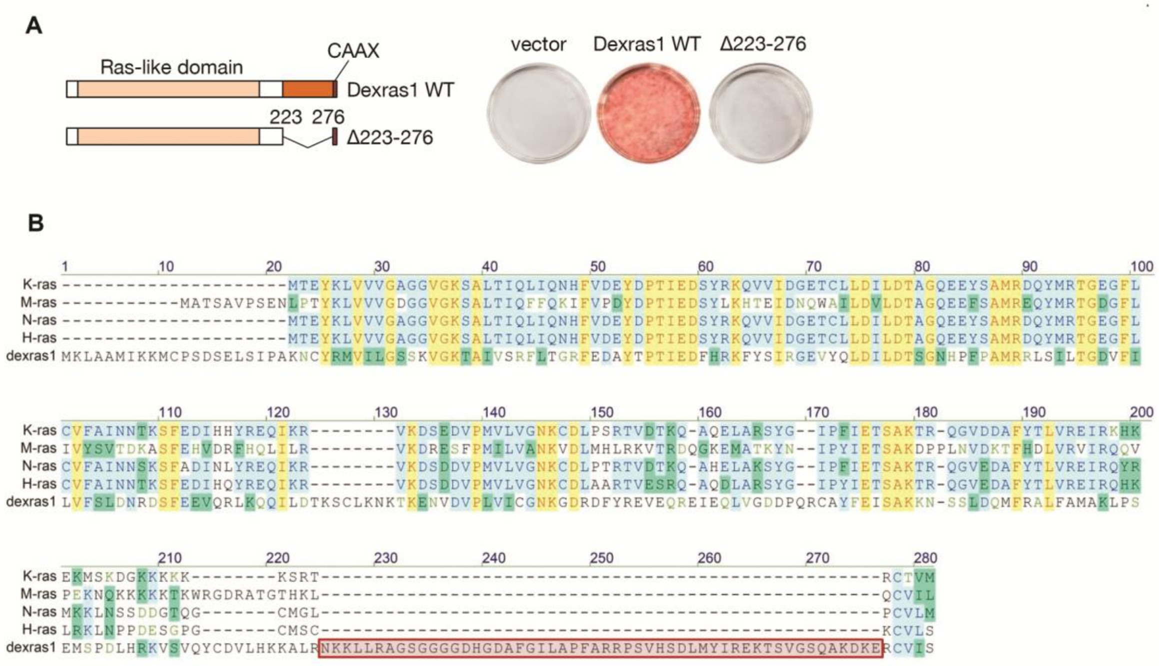

Supplementary Figure S3. The C-terminal domain is important for Dexras1 function. **(A)** Schematic drawing of wild type and mutant of Dexras1 used in this study (left). C-terminal four amino acids, correspond to the canonic CAAX (where C indicates cysteine; A, aliphatic; and X, any amino acids) were marked. Oil-red-O staining of differentiated 3T3-L1 cells by IBMX and insulin (right). **(B)** Comparison of amino acid sequences between ras family and Dexras1. Murine K-ras, M-ras, N-ras, H-ras, and Dexras1 amino acid sequences were aligned using VectorNTI program. The C-terminal domain of Dexras1, which is not found in other ras family genes, is marked in the red box. Sequence positives = 61.2%, sequence identity = 15.3%.

Supplementary Figure S4

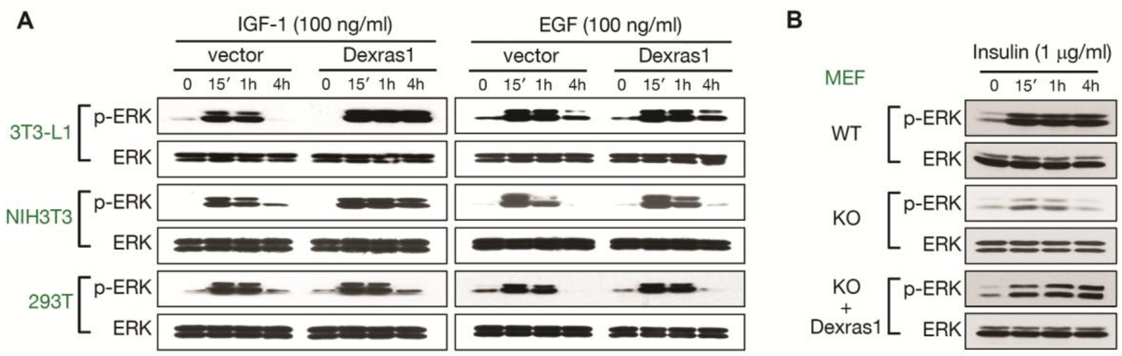

Supplementary Figure S4. Dexras1 activates ERK during adipogenesis in response to insulin/IGF-1 but not by EGF. **(A)** 3T3-L1, NIH3T3, or 293T cells were transfected with empty vector or the Dexras1-FLAG expression construct, and then stimulated with IGF-1 or EGF. The total and phosphorylated ERK were detected by western blot at indicated time points. **(B)** Overexpression of Dexras1 rescues insulin-induced ERK activation in knockout MEFs. WT or Dexras1 KO MEFs were transfected with pcDNA3 vector or pcDNA3-Dexras1-FLAG and subjected to differentiation with IBMX, dexamethasone, and insulin (1 μg/ml) in the presence of rosiglitasone.

Supplementary Figure S5

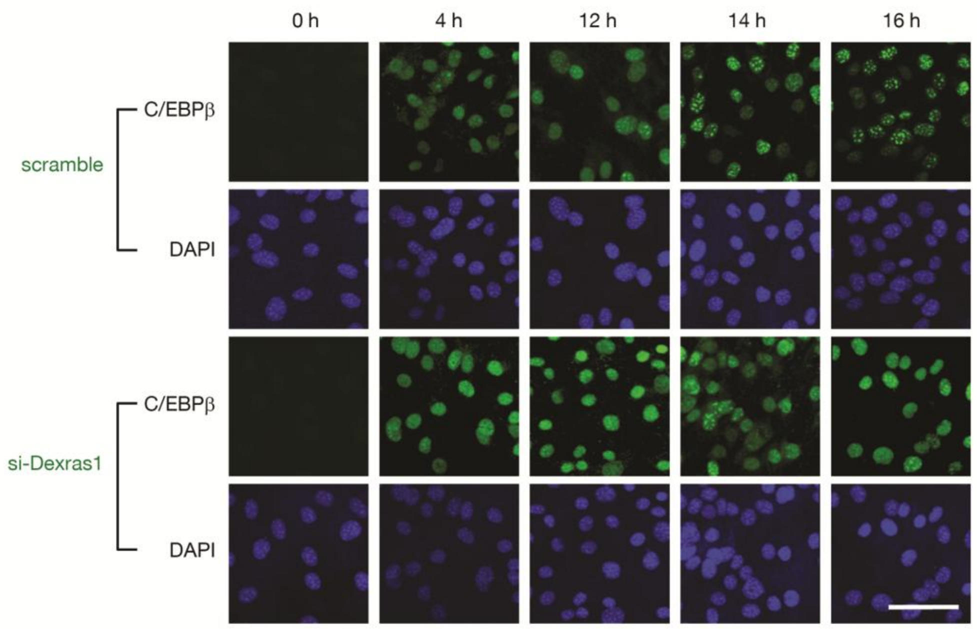

Supplementary Figure S5. Immunofluorescence study of C/EBPβ during adipogenesis. 3T3-L1 preadipocytes were induced to differentiate with the standard protocol with either scramble or si-Dexras1. At indicated time points, cells were fixed and subjected to immunofluorescence analysis with antibody against C/EBPβ and 4',6-diamidino-2-phenylindole. Fluorescence images were obtained by confocal microscopy. Scale bar = 50 μm.

Supplementary Figure S6

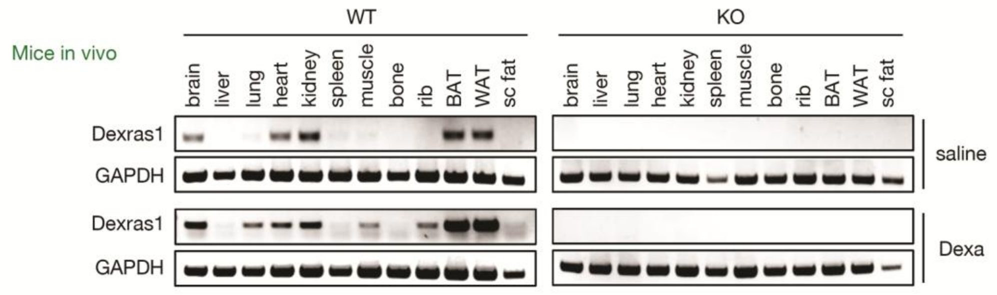

Supplementary Figure S6. Dexras1 expression in response to dexamethasone treatment in wild type and Dexras1 knockout (KO) mice. Wild type and Dexras1 knockout (KO) mice were subjected to dexamethasone treatment (10 mg/kg) for 3 months by intraperitoneal injection, three times a week. Mice were sacrificed and Dexras1 expression was measured by RT-PCR.

Supplementary Figure S7

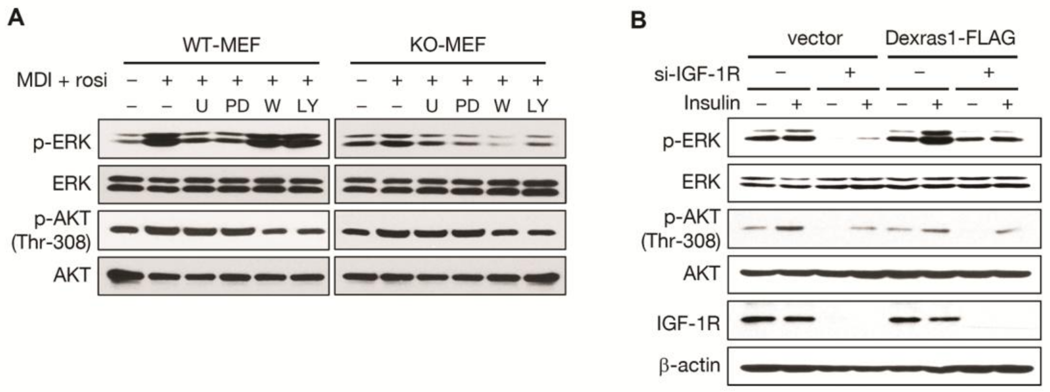

Supplementary Figure S7. Dexras1 is required for ERK activation, but not AKT activation, and is dependent on IGF-1R. **(A)** Wild type or Dexras1 KO MEF cells were pre-treated with the indicated reagents (U, U0126; PD, PD98059; W, wortmannin; or LY, LY294002). After 30 minutes, cells were differentiated with IBMX, dexamethasone, and insulin (MDI) in the presence of rosiglitasone. ERK and AKT activation was evaluated by immunoblot for phosphorylated versions of these proteins. **(B)** 3T3-L1 cells were transfected with pcDNA3 vector or pcDNA3-Dexras1-FLAG and treated with scramble or IGF-1R siRNAs. Cells were incubated with insulin (1 μg/ml) for 1 h and subjected to immunoblot analysis.
